# Research into Policy: A Brief History of Mitochondrial Donation

**DOI:** 10.1002/stem.2221

**Published:** 2015-10-27

**Authors:** Lyndsey Craven, Mary Herbert, Alison Murdoch, Julie Murphy, James Lawford Davies, Doug M. Turnbull

**Affiliations:** ^1^ Wellcome Trust Centre for Mitochondrial Research, Institute of NeuroscienceNewcastle UniversityNewcastle upon TyneUnited Kingdom; ^2^ Wellcome Trust Centre for Mitochondrial Research, Institute of Genetic Medicine, Newcastle UniversityNewcastle upon TyneUnited Kingdom; ^3^ Newcastle Fertility Centre, Newcastle Hospitals NHS Foundation TrustNewcastle upon TyneUnited Kingdom; ^4^ HempsonsHempsons House, Villiers StreetLondonUnited Kingdom

**Significance Statement**

After 17 years of extensive debate and discussion, mitochondrial donation may now, for the first time, give some women with mitochondrial DNA disease the opportunity to have a healthy child that is genetically related to both parents.

Mitochondrial DNA disorders are a group of common genetic diseases which affect both children and adults. They lead to progressive multisystem disease for which there is no curative treatment. Inherited mitochondrial DNA mutations are transmitted maternally, and preventing transmission of these diseases is a priority for families. An important new approach is a novel *in vitro* fertilisation (IVF) technique called mitochondrial donation using either maternal spindle transfer or pronuclear transfer 1. The 4th March 2015 saw an historic event—the legislation was put in place to make mitochondrial donation legal in the UK. Critics of the technique have claimed that the new law was rushed through and that there had been insufficient time to debate the issues. Here we provide a brief account of the past 17 years to show that this is not the case.

The complete human mitochondrial genome, made up of only 16,569 base pairs, was first sequenced in 1981 2. Around the same time, in an entirely unrelated area of research, the technique of nuclear transplantation between mouse embryos was described 3, 4. The first human pathogenic mitochondrial DNA mutations were identified in 1988 5, 6 and by 1995, the possibility of preventing the transmission of mitochondrial DNA disease by nuclear transplantation was already being considered 7. Since then, although the name for the technique we now know as mitochondrial donation has changed several times, the scientific, ethical, and legal issues have been examined in detail by a number of independent groups and committees over many years.

The need to regulate the use of human embryos in both fertility treatment and scientific research was recognized in the UK following the birth of the first IVF baby in 1978. This major breakthrough led to concerns about the social and legal implications of such advances in human assisted reproduction. To address these issues, the Government established the Committee of Inquiry into Human Fertilisation and Embryology chaired by the now Baroness Mary Warnock. The report 8, published in 1984, set out a blue‐print for the regulation of both IVF and embryo research, and with admirable foresight, included a chapter describing possible future developments in embryo research. This report was followed by the White Paper “Human Fertilisation and Embryology: A Framework for Legislation” which was published in 1987 and formed the basis for the Human Fertilisation and Embryology Act 1990 (“the 1990 Act”) 9.

There were rapid and significant advances in non‐human embryo research over subsequent years, including somatic cell nuclear transfer and the birth of Dolly the sheep in 1996 10. These developments generated much public interest and highlighted the need for a clarification of the legislation regarding the implications for human clinical embryology. A report by the Human Fertilisation and Embryology Authority (HFEA) and Human Genetics Advisory Commission (HGAC) 11, published in 1998, acknowledged that some of the scientific possibilities being discussed at this time had not been envisaged when the 1990 Act was drafted. Specifically, the Report recommended that the purposes for which human embryos could be used in research should be extended to allow the development of methods of therapy for mitochondrial diseases.

Following this Report, the Government established an expert group chaired by the Chief Medical Officer (Professor Sir Liam Donaldson) to examine the potential benefits of a number of new areas of human embryo research, including methods to prevent mitochondrial disease. The report 12 made several recommendations including research to increase the understanding of, and develop treatments for, mitochondrial disease.

The Human Fertilisation and Embryology (Research Purposes) Regulations 2001 followed 13, which extended the purposes for which research on early human embryos could be undertaken. A Select Committee on Stem Cell Research was appointed to consider the issues arising from these regulations. The report 14, published in 2002, stated that the methods to prevent mitochondrial disease could have “great potential” and that there was a “strong scientific and medical case for further research.”

In May 2004, our team at Newcastle University applied for a research licence from the HFEA to conduct research into using pronuclear transfer to avoid the transmission of mitochondrial disease. After extensive review of both the scientific methodology and the legal implications of this proposal by the HFEA, the team was granted a licence in September 2005.

In 2008, thorough revisions to the 1990 Act were passed by a significant majority in both Houses of Parliament following nearly 2 years of debate. This included a specific provision to allow for regulations to be passed in the future by Parliament to permit the clinical application of “techniques that alter the DNA of an egg or embryo to prevent the transmission of serious mitochondrial disease” (section 3ZA(5)) 15.

In May 2010, the HFEA's Scientific and Clinical Advances Advisory Committee (“SCAAC”) considered the potential use of mitochondrial donation to avoid the transmission of mitochondrial disease. It was then in November 2010 that the Government was invited to consider exercising the regulation‐making power added to the 1990 Act in order to make it possible for mitochondrial donation to be used as a clinical treatment. Following this request, in February 2011 the Secretary of State for Health asked the HFEA to scope “expert views on the effectiveness and safety of mitochondrial transfer.” The HFEA established an independent panel to collate and summarize scientific evidence from a wide range of experts in the field. The panel published its first report in April 2011 16 and subsequently met and published two further reports in March 2013 17 and June 2014 18.

Many important scientific developments were considered by the panel in these reports, including that maternal spindle transfer between non‐human primate oocytes resulted in the birth of healthy offspring with the capacity to prevent mitochondrial DNA disease 19. Data published by our group demonstrated that pronuclear transfer between human embryos was feasible and had the potential to prevent mitochondrial DNA disease 20. Further experiments performed using human oocytes confirmed that maternal spindle transfer had the potential to prevent mitochondrial DNA disease 21, 22 but required further optimization to improve the efficiency of the technique. Following careful consideration of the data provided by these studies, and many others, the panel reached the same conclusion following each report that there was no evidence to suggest that the technique of mitochondrial donation was unsafe for clinical use.

The ethical concerns about using this technology in humans were debated widely. An independent public consultation and review by the Nuffield Council on Bioethics considered the implications of the proposed techniques. Their report 23 was published in June 2012 and supported the clinical use of mitochondrial donation.

The HFEA were also asked to seek public views on mitochondrial donation and a public dialogue launched in collaboration with Sciencewise in December 2012 24. The consultation was thoroughly evaluated and credited as an exemplary exercise in public engagement for policy purposes 25. The HFEA published its report of the consultation in March 2013 26 and its advice to Government was that there was general support for permitting mitochondrial donation in the UK. It was also reported that the ethical concerns were outweighed by the arguments in favor of permitting mitochondrial donation.

In June 2013, following the collective outcomes of the scientific reviews, the public consultation and the report of the Nuffield Council on Bioethics, the UK Government announced their decision to publish draft regulations to amend the 1990 Act to permit the clinical application of mitochondrial donation. The draft regulations were published in February 2014 and a further public consultation launched 27. Following careful consideration of the responses to this consultation, regulations were laid before Parliament in December 2014.

On 3 February 2015, there was a debate and vote in the House of Commons to decide whether to approve the draft Regulations to allow mitochondrial donation. The House of Commons voted by 382 to 128 in support of the Regulations. Three weeks later, on 24 February 2015, the House of Lords debated the draft Regulations and voted by 280 to 48 in support, allowing the Regulations to become law.

On 4 March 2015, the Parliamentary Under‐Secretary of State for Public Health at the Department of Health (Jane Ellison MP) signed the Mitochondrial Donation Regulations 28, making mitochondrial donation legal for the first time in the UK. The HFEA will now develop a licensing framework through which applications can be considered on a case by case basis. After 17 years of extensive debate and discussion, mitochondrial donation may now, for the first time, give some women with mitochondrial DNA disease the opportunity to have a healthy child that is genetically related to both parents. While the UK is the first to legislate in this area, there is likely to be interest from other countries in mitochondrial donation. For example, the US Food and Drug Administration has requested that the Institute of Medicine produce a consensus report into the ethical and social policy considerations of novel techniques for prevention of maternal transmission of mitochondrial DNA diseases.

## Acknowledgments

The authors work was funded by The Wellcome Trust Centre for Mitochondrial Research [G096919], Newcastle University Centre for Brain Ageing and Vitality (supported by the Biotechnology and Biological Sciences Research Council, Engineering and Physical Sciences Research Council, Economic and Social Research Council and Medical Research Council [G0700718]), MRC Centre for Neuromuscular Disease [G000608‐1], The MRC Centre for Translational Research in Neuromuscular Disease Mitochondrial Disease Patient Cohort (UK) [G0800674], Lily Foundation, the UK NIHR Biomedical Research Centre in Age and Age Related Diseases award to the Newcastle upon Tyne Hospitals NHS Foundation Trust and UK NHS Specialist Commissioners “Rare Mitochondrial Disorders of Adults and Children” Service.

## Author Contributions

All authors contributed to the drafting of the manuscript and approved the final version.

## Disclosure of Potential Conflicts of Interest

The authors indicate no potential conflicts of interest.

## References

[1] Richardson J , Irving L , Hyslop LA et al. Concise reviews: Assisted reproductive technologies to prevent transmission of mitochondrial DNA disease. Stem Cells 2015;33:639–45. 25377180 10.1002/stem.1887 PMC4359624

[2] Anderson S , Bankier AT , Barrell BG et al. Sequence and organization of the human mitochondrial genome. Nature 1981;290:457–65. 7219534 10.1038/290457a0

[3] Illmensee K , Hoppe PC . Nuclear transplantation in *Mus musculus*: Developmental potential of nuclei from preimplantation embryos. Cell 1981;23:9–18. 7214529 10.1016/0092-8674(81)90265-8

[4] McGrath J , Solter D . Nuclear transplantation in the mouse embryo by microsurgery and cell fusion. Science 183;220:1300–1302. 6857250 10.1126/science.6857250

[5] Holt IJ , Harding AE , Morgan‐Hughes JA . Deletions of muscle mitochondrial DNA in patients with mitochondrial myopathies. Nature 1988;331:717–719. 2830540 10.1038/331717a0

[6] Wallace DC , Singh G , Lott MT et al. Mitochondrial DNA mutation associated with Leber's hereditary optic neuropathy. Science 1988;242:1427–30. 3201231 10.1126/science.3201231

[7] Rubenstein DS , Thomasma DC , Schon EA et al. Germ‐line therapy to cure mitochondrial disease: Protocol and ethics of in vitro ovum nuclear transplantation. Camb Q Healthc Ethics 1995;4:316–339. 7551145 10.1017/s0963180100006071

[8] Report of the Committee of Inquiry into Human Fertilisation and Embryology. Available at http://www.hfea.gov.uk/2068.html, 1984. 3846590

[9] Human Fertilisation and Embryology Act . Available at http://www.legislation.gov.uk/ukpga/1990/37/contents, 1990.

[10] Wilmut I , Schnieke AE , McWhir J et al. Viable offspring derived from fetal and adult mammalian cells. Nature 1997;385:810–813. 9039911 10.1038/385810a0

[11] Cloning Issues in Reproduction, Science and Medicine . Available at http://www.hfea.gov.uk/docs/Cloning_Issue_Report.pdf, 1998.

[12] Stem Cell Research: Medical Progress with Responsibility . Available at http://webarchive.nationalarchives.gov.uk/20130107105354/http:// www.dh.gov.uk/prod_consum_dh/groups/dh_ digitalassets/@dh/@en/documents/digitalasset/dh_4065085.pdf, 2000.

[13] The Human Fertilisation and Embryology (Research Purposes) Regulations . Available at http://www.legislation.gov.uk/uksi/2001/188/contents/made, 2001.

[14] House of Lords, Stem Cell Research—Report. Available at http://www.parliament.the-stationery-office.co.uk/pa/ld200102/ldselect/ldstem/83/8301.htm, 2002.

[15] Human Fertilisation and Embryology Act . Available at http://www.legislation.gov.uk/ukpga/2008/22/contents, 2008.

[16] Scientific review of the safety and efficacy of methods to avoid mitochondrial disease through assisted conception. http://www.hfea.gov.uk/docs/2011-04-18_Mitochondria_review_-_final_report.PDF, April 2011.

[17] Scientific review of the safety and efficacy of methods to avoid mitochondrial disease through assisted conception: Update. Available at http://www.hfea.gov.uk/docs/Mito-Annex_VIII-science_review_update.pdf, March 2013.

[18] Third scientific review of the safety and efficacy of methods to avoid mitochondrial disease through assisted conception: 2014 update. Available at http://www.hfea.gov.uk/docs/Third_Mitochondrial_replacement_scientific_ review.pdf, June 2014.

[19] Tachibana M , Sparman M , Sritanaudomchai H et al. Mitochondrial gene replacement in primate offspring and embryonic stem cells. Nature 2009;461:367–372. 19710649 10.1038/nature08368 PMC2774772

[20] Craven L , Tuppen HA , Greggains GD et al. Pronuclear transfer in human embryos to prevent transmission of mitochondrial DNA disease. Nature 2010;465:82–85. 20393463 10.1038/nature08958 PMC2875160

[21] Tachibana M , Amato P , Sparman M et al. Towards germline gene therapy of inherited mitochondrial diseases. Nature 2013;493:627–631. 23103867 10.1038/nature11647 PMC3561483

[22] Paull D , Emmanuele V , Weiss KA et al. Nuclear genome transfer in human oocytes eliminates mitochondrial DNA variants. Nature 2013;493:632–637. 23254936 10.1038/nature11800 PMC7924261

[23] Novel techniques for the prevention of mitochondrial DNA disorders: An ethical review, Nuffield Council on Bioethics. Available at http://nuffieldbioethics.org/wp‐content/uploads/2014/06/Novel_techniques_for_the_prevention_of_mitochondrial_DNA_disorders_compressed.pdf, 2012.

[24] Medical frontiers: Debating mitochondria replacement. Available at http://www.hfea.gov.uk/9359.html, 2012.

[25] Mitochondria Replacement, Sciencewise. Available at http://www.sciencewise-erc.org.uk/cms/assets/Uploads/Mitochondria-evaluation-FINAL-2013.pdf, 2012.

[26] Mitochondria replacement consultation: Advice to Government. http://www.hfea.gov.uk/docs/Mitochondria_replacement_consultation_-_advice_for_Government.pdf, Accessed March 2013.

[27] Mitochondrial Donation. Available at https:// www.gov.uk/government/uploads/system/uploads/attachment_data/file/332881/Consultation_response.pdf. Accessed July 2014

[28] The Human Fertilisation and Embryology (Mitochondrial Donation) Regulations. Available at http://www.legislation.gov.uk/uksi/2015/572/contents/made, 2015.

